# Consequences of COVID-19 and Its Variants: Understanding the Physical, Oral, and Psychological Impact

**DOI:** 10.3390/ijerph20043099

**Published:** 2023-02-10

**Authors:** Kelvin I. Afrashtehfar, Carlos A. Jurado, Amaweya Al-Sammarraie, Musab H. Saeed

**Affiliations:** 1 Division of Restorative Dental Sciences, Clinical Sciences Department, Ajman College of Dentistry, Ajman City P.O. Box 346, United Arab Emirates; 2 Department of Reconstructive Dentistry and Gerodontology, School of Dental Medicine, University of Bern, 3010 Bern, Switzerland; 3 Department of Prosthodontics, The University of Iowa College of Dentistry and Dental Clinics, Iowa City, IA 52242, USA

## 1. Background

The highly infectious severe acute respiratory syndrome coronavirus-2 (SARS-CoV-2) caused the coronavirus disease 2019 (COVID-19) pandemic, which affects the lives of people worldwide in a variety of unprecedented ways. The virus not only causes severe respiratory illness but also significantly impacts physical, oral, and psychological health [1,2,3,4,5,6]. As the pandemic continues to evolve, it is crucial to understand the long-term consequences of COVID-19 on overall human well-being. The purpose of this editorial is to provide an overview of the current research into the physical, oral, and psychological sequelae of COVID-19 and to highlight the importance of addressing these issues in the ongoing fight against the virus.

According to the World Health Organization (WHO), COVID-19 can cause various symptoms, including fever, cough, and a difficulty breathing [7]. The virus can also lead to severe illness and death, particularly in older adults and people with underlying health conditions [1,8,9]. In addition to the physical symptoms, COVID-19 affects oral health, with dry mouth, tooth decay, and periodontal disease among the reported consequences [4,10,11]. Furthermore, the pandemic has also significantly impacted mental health, with the isolation and uncertainty caused by the virus leading to increased rates of stress, anxiety, and depression [5,6,12,13].

In this editorial, we will discuss the physical, oral, and psychological consequences of COVID-19. We will also explore the pandemic’s challenges and opportunities in addressing these consequences and maintaining overall health and well-being.

## 2. Physical Consequences of COVID-19

The most common physical symptoms of COVID-19 include fever, cough, and difficulty breathing. These symptoms typically appear 2–14 days after exposure to the virus [14]. In addition to these symptoms, other common physical challenges of COVID-19 include fatigue, muscle aches, sore throat, headache, loss of smell or taste, and conjunctivitis [15].

In addition to these acute symptoms, there can also be long-term physical side effects to being infected with COVID-19. Around one-third of hospitalized COVID-19 patients continue to experience symptoms such as fatigue, muscle weakness, and breathlessness for several weeks or months after their initial illness [16]. Another study [17] reported that a significant proportion of COVID-19 patients developed acute respiratory distress syndrome, a pathology which can lead to chronic lung damage. Additionally, there have been reports of COVID-19 patients developing organ damage, such as myocarditis and pericarditis [18]. Studies have found that a good proportion of the COVID-19 patients who were discharged from the hospital were found to have abnormal results on echocardiography, an ultrasound of the heart, thus indicating that COVID-19 may lead to heart damage in some patients [19]. In addition, COVID-19 patients are at increased risk of developing chronic fatigue syndrome, a condition characterized by severe fatigue that is not alleviated by rest and which persists for several months [20,21].

In summary, COVID-19 can cause a wide range of physical symptoms, from common symptoms like fever, cough, and difficulty breathing to long-term problems such as chronic fatigue, organ damage, and chronic lung damage. These findings highlight the importance of continued research and monitoring of the physical effects of COVID-19, as well as the need to provide appropriate care and support for patients experiencing long-term physical symptoms.

## 3. Oral Consequences of COVID-19

COVID-19 can significantly impact oral health, with several studies reporting an association between COVID-19 infection and oral symptoms such as dry mouth, tooth decay, and periodontal disease [4,10,11]. Indeed, this was confirmed by studies that showed COVID-19 patients to have a significantly higher prevalence of oral symptoms such as dry mouth, toothache, and bad taste compared to healthy controls [22]. Additionally, COVID-19 patients were found to have a higher prevalence of dental caries and periodontitis than healthy controls [23].

The pandemic has also affected oral healthcare access and delivery, with many dental clinics and practices closing or reducing services due to the risk of viral transmission. According to an American study [24], dental visits decreased by more than 20% during the early months of the pandemic. This reduction in access to oral health care had a detrimental effect on oral health, particularly for individuals with pre-existing oral health conditions.

Recent data on the oral consequences of COVID-19 have shown a link between infection with the virus and oral complications. A narrative review concluded that COVID-19 patients had a higher prevalence of oral manifestations compared to healthy controls [25]. A separate Nigerian study [26] found that COVID-19 patients had a higher prevalence of oral complications such as dry mouth, taste disturbances, and oral ulcers than healthy controls.

To sum up, COVID-19 and its variants can significantly impact oral health, with studies reporting an association between infection and oral symptoms such as dry mouth, tooth decay, and periodontal disease. The pandemic has also affected oral healthcare access and delivery, with many dental clinics and practices closing or reducing services. With the emergence of new variants of SARS-CoV-2, it is crucial to monitor the oral consequences of the disease and consider the oral health of patients infected with COVID-19.

## 4. Psychological Consequences of COVID-19

The COVID-19 pandemic has had a significant psychological impact on individuals worldwide, leading to increased levels of stress, anxiety, and depression, with those who have been infected with the virus or have had close contact with infected individuals being at a higher risk [2,5,6,12,13].

The isolation and uncertainty brought about by the pandemic have also significantly impacted mental health. Individuals who experienced isolation and uncertainty during the pandemic had higher levels of anxiety and depression compared to those who did not [27,28]. The quarantine measures and social distancing have also been linked to increased feelings of loneliness and social isolation, which can further exacerbate mental health problems [29].

To cope with the psychological consequences of COVID-19, individuals can take a variety of measures, such as staying connected with loved ones, engaging in regular physical activity, and practicing mindfulness and relaxation techniques [30]. Psychological support and counseling can also help to manage stress and anxiety [5]. Additionally, seeking professional help in case of signs of mental health issues such as depression is essential [31]. Additionally, it is crucial to follow guidelines provided by health authorities regarding the disease and lockdown measures, and also to stay informed and educated about the disease and its evolution.

In summary, the COVID-19 pandemic has had a significant psychological impact on individuals worldwide, with increased levels of stress, anxiety, and depression being reported. The isolation and uncertainty brought about by the pandemic have also significantly impacted mental health. To cope with these repercussions, individuals must take proactive measures such as staying connected with loved ones, engaging in regular physical activity, and practicing mindfulness and relaxation techniques. Additionally, seeking professional help and following guidelines provided by health authorities can also help manage the psychological consequences of the pandemic.

## 5. Conclusions

The COVID-19 pandemic has significantly impacted various aspects of human health, including physical, oral, and psychological well-being. The most common physical symptoms of COVID-19 include fever, cough, and difficulty breathing, while long-term consequences include chronic fatigue and organ damage. The pandemic has also affected oral health, with symptoms such as dry mouth, tooth decay, and periodontal disease being reported. Moreover, the pandemic has significantly impacted mental health, leading to increased stress, anxiety, and depression. Therefore, scientists and policymakers need to understand the physical, oral, and psychological consequences of COVID-19 in order to conduct further research that can inform public health policies and interventions to mitigate the adverse effects of the pandemic on human health. For example, researching the impact of the pandemic on oral health care access and delivery can inform policies that aim to improve oral health care during the pandemic. Additionally, research on the psychological impact of the pandemic on different groups of people, such as healthcare workers and essential workers, can inform interventions that aim to support mental well-being. Moreover, it is vital to keep in mind the guidelines provided by health authorities, to follow them and protect oneself and others from the disease, and to continue to monitor the evolution of the disease to provide updated information for the public and research purposes.

In a nutshell, this research editorial aimed to provide an overview of the physical, oral, and psychological sequalae of COVID-19 and its variants, as well as to highlight the importance of understanding these consequences for further research. Scientists should consider health authority guidelines and continue monitoring the disease’s evolution. This will help to inform public health policies and interventions that aim to mitigate the adverse effects of the pandemic on human health.
